# Supplementary material for: Modeling glioblastoma heterogeneity as a dynamic network of cell states
Source: Mol Syst Biol. 2021 Sep 16;17(9):e10105. doi: 10.15252/msb.202010105 (PMC8444284; doi:10.15252/msb.202010105)
Supplement: Supplementary file 6 — Source Data for Figure 5 [file MSB-17-e10105-s004.zip › Figure5A_sourcedata/GSEA_3071/nosigdb_stateV.GseaPreranked.1622795159954/DOROSZKO_U3054MG_CLUSTER_4.html]

Details for gene set DOROSZKO\_U3054MG\_CLUSTER\_4[GSEA]

|  || Dataset | state43071 |
| Phenotype | NoPhenotypeAvailable |
| Upregulated in class | na\_pos |
| GeneSet | DOROSZKO\_U3054MG\_CLUSTER\_4 |
| Enrichment Score (ES) | 0.65940297 |
| Normalized Enrichment Score (NES) | 4.542767 |
| Nominal p-value | 0.0 |
| FDR q-value | 0.0 |
| FWER p-Value | 0.0 |
Table: GSEA Results Summary

  

Fig 1: Enrichment plot: DOROSZKO\_U3054MG\_CLUSTER\_4      
 Profile of the Running ES Score & Positions of GeneSet Members on the Rank Ordered List

  

| PROBE | GENE SYMBOL | GENE\_TITLE | RANK IN GENE LIST | RANK METRIC SCORE | RUNNING ES | CORE ENRICHMENT || 1 | HIST1H1B |  |  | 0 | 2.500 | 0.0059 | Yes |
| 2 | HIST1H4C |  |  | 1 | 2.442 | 0.0117 | Yes |
| 3 | ATAD2 |  |  | 2 | 2.214 | 0.0170 | Yes |
| 4 | ESCO2 |  |  | 3 | 2.193 | 0.0222 | Yes |
| 5 | FAM111B |  |  | 4 | 2.012 | 0.0270 | Yes |
| 6 | HMGB2 |  |  | 5 | 2.011 | 0.0317 | Yes |
| 7 | NUSAP1 |  |  | 6 | 2.001 | 0.0365 | Yes |
| 8 | PCNA |  |  | 10 | 1.908 | 0.0404 | Yes |
| 9 | DRAXIN |  |  | 12 | 1.882 | 0.0446 | Yes |
| 10 | TOP2A |  |  | 14 | 1.842 | 0.0488 | Yes |
| 11 | HIST1H1E |  |  | 15 | 1.830 | 0.0531 | Yes |
| 12 | ASRGL1 |  |  | 16 | 1.819 | 0.0575 | Yes |
| 13 | MCM4 |  |  | 17 | 1.815 | 0.0618 | Yes |
| 14 | DHFR |  |  | 18 | 1.795 | 0.0660 | Yes |
| 15 | HIST1H1D |  |  | 19 | 1.786 | 0.0703 | Yes |
| 16 | C21orf58 |  |  | 20 | 1.772 | 0.0745 | Yes |
| 17 | NCAPG |  |  | 21 | 1.760 | 0.0786 | Yes |
| 18 | RAD51AP1 |  |  | 22 | 1.757 | 0.0828 | Yes |
| 19 | FAM111A |  |  | 24 | 1.738 | 0.0867 | Yes |
| 20 | SYNE2 |  |  | 25 | 1.732 | 0.0908 | Yes |
| 21 | H2AFX |  |  | 26 | 1.725 | 0.0949 | Yes |
| 22 | PCLAF |  |  | 27 | 1.720 | 0.0990 | Yes |
| 23 | CENPF |  |  | 28 | 1.705 | 0.1030 | Yes |
| 24 | TYMS |  |  | 30 | 1.683 | 0.1068 | Yes |
| 25 | CCNE2 |  |  | 32 | 1.664 | 0.1106 | Yes |
| 26 | RRM2 |  |  | 34 | 1.660 | 0.1143 | Yes |
| 27 | MYBL2 |  |  | 35 | 1.651 | 0.1182 | Yes |
| 28 | CENPU |  |  | 36 | 1.645 | 0.1221 | Yes |
| 29 | KNL1 |  |  | 38 | 1.629 | 0.1258 | Yes |
| 30 | CDCA7L |  |  | 39 | 1.619 | 0.1296 | Yes |
| 31 | SGO1 |  |  | 40 | 1.614 | 0.1334 | Yes |
| 32 | E2F1 |  |  | 44 | 1.602 | 0.1366 | Yes |
| 33 | MKI67 |  |  | 45 | 1.598 | 0.1404 | Yes |
| 34 | BRCA1 |  |  | 46 | 1.592 | 0.1442 | Yes |
| 35 | HELLS |  |  | 48 | 1.588 | 0.1477 | Yes |
| 36 | MCM3 |  |  | 49 | 1.586 | 0.1515 | Yes |
| 37 | XRCC2 |  |  | 50 | 1.574 | 0.1552 | Yes |
| 38 | KIF15 |  |  | 52 | 1.568 | 0.1587 | Yes |
| 39 | FOXM1 |  |  | 53 | 1.550 | 0.1624 | Yes |
| 40 | PBK |  |  | 54 | 1.536 | 0.1661 | Yes |
| 41 | EZH2 |  |  | 55 | 1.534 | 0.1697 | Yes |
| 42 | GINS2 |  |  | 57 | 1.528 | 0.1731 | Yes |
| 43 | EMP2 |  |  | 59 | 1.511 | 0.1765 | Yes |
| 44 | DTL |  |  | 60 | 1.503 | 0.1801 | Yes |
| 45 | MXD3 |  |  | 62 | 1.500 | 0.1834 | Yes |
| 46 | GTSE1 |  |  | 66 | 1.491 | 0.1863 | Yes |
| 47 | E2F8 |  |  | 67 | 1.488 | 0.1899 | Yes |
| 48 | CDK1 |  |  | 69 | 1.488 | 0.1932 | Yes |
| 49 | SMC2 |  |  | 73 | 1.466 | 0.1960 | Yes |
| 50 | SPC25 |  |  | 76 | 1.442 | 0.1990 | Yes |
| 51 | CHAF1A |  |  | 77 | 1.441 | 0.2025 | Yes |
| 52 | UBE2T |  |  | 79 | 1.432 | 0.2056 | Yes |
| 53 | FANCD2 |  |  | 81 | 1.423 | 0.2088 | Yes |
| 54 | FBXO5 |  |  | 82 | 1.422 | 0.2122 | Yes |
| 55 | KLHL23 |  |  | 83 | 1.421 | 0.2156 | Yes |
| 56 | CLSPN |  |  | 84 | 1.417 | 0.2189 | Yes |
| 57 | CIP2A |  |  | 86 | 1.410 | 0.2221 | Yes |
| 58 | MCM7 |  |  | 88 | 1.400 | 0.2252 | Yes |
| 59 | MCM8 |  |  | 89 | 1.397 | 0.2285 | Yes |
| 60 | HES6 |  |  | 91 | 1.391 | 0.2316 | Yes |
| 61 | MCM10 |  |  | 92 | 1.389 | 0.2349 | Yes |
| 62 | FBLN1 |  |  | 95 | 1.383 | 0.2377 | Yes |
| 63 | RRM1 |  |  | 99 | 1.364 | 0.2404 | Yes |
| 64 | CARHSP1 |  |  | 101 | 1.362 | 0.2434 | Yes |
| 65 | OLFM1 |  |  | 102 | 1.361 | 0.2466 | Yes |
| 66 | TOPBP1 |  |  | 108 | 1.350 | 0.2488 | Yes |
| 67 | POLD3 |  |  | 111 | 1.340 | 0.2515 | Yes |
| 68 | KIFC1 |  |  | 113 | 1.338 | 0.2545 | Yes |
| 69 | CENPM |  |  | 114 | 1.337 | 0.2577 | Yes |
| 70 | TACC3 |  |  | 116 | 1.334 | 0.2606 | Yes |
| 71 | CDCA5 |  |  | 123 | 1.326 | 0.2625 | Yes |
| 72 | NSD2 |  |  | 124 | 1.325 | 0.2657 | Yes |
| 73 | SMC4 |  |  | 127 | 1.320 | 0.2684 | Yes |
| 74 | BARD1 |  |  | 132 | 1.310 | 0.2707 | Yes |
| 75 | MMS22L |  |  | 134 | 1.306 | 0.2735 | Yes |
| 76 | FEN1 |  |  | 139 | 1.296 | 0.2758 | Yes |
| 77 | RTKN2 |  |  | 143 | 1.285 | 0.2782 | Yes |
| 78 | LIG1 |  |  | 145 | 1.284 | 0.2811 | Yes |
| 79 | GMNN |  |  | 146 | 1.280 | 0.2841 | Yes |
| 80 | NCAPD3 |  |  | 155 | 1.261 | 0.2854 | Yes |
| 81 | NCAPD2 |  |  | 157 | 1.252 | 0.2882 | Yes |
| 82 | MIS18BP1 |  |  | 159 | 1.247 | 0.2909 | Yes |
| 83 | POLQ |  |  | 160 | 1.245 | 0.2939 | Yes |
| 84 | TMPO |  |  | 161 | 1.243 | 0.2968 | Yes |
| 85 | CENPK |  |  | 162 | 1.241 | 0.2998 | Yes |
| 86 | CDKN3 |  |  | 163 | 1.239 | 0.3027 | Yes |
| 87 | LMNB1 |  |  | 165 | 1.238 | 0.3054 | Yes |
| 88 | DNMT1 |  |  | 166 | 1.237 | 0.3084 | Yes |
| 89 | KIF23 |  |  | 167 | 1.235 | 0.3113 | Yes |
| 90 | NCAPH |  |  | 168 | 1.235 | 0.3142 | Yes |
| 91 | CDCA3 |  |  | 169 | 1.230 | 0.3172 | Yes |
| 92 | RFC4 |  |  | 171 | 1.227 | 0.3199 | Yes |
| 93 | RNF168 |  |  | 175 | 1.225 | 0.3221 | Yes |
| 94 | CCDC14 |  |  | 179 | 1.214 | 0.3244 | Yes |
| 95 | NASP |  |  | 180 | 1.212 | 0.3273 | Yes |
| 96 | GGH |  |  | 182 | 1.207 | 0.3299 | Yes |
| 97 | DBF4 |  |  | 189 | 1.197 | 0.3315 | Yes |
| 98 | KIF4A |  |  | 191 | 1.193 | 0.3341 | Yes |
| 99 | NUF2 |  |  | 192 | 1.189 | 0.3370 | Yes |
| 100 | CDT1 |  |  | 194 | 1.185 | 0.3396 | Yes |
| 101 | PXMP2 |  |  | 195 | 1.180 | 0.3424 | Yes |
| 102 | FANCI |  |  | 196 | 1.179 | 0.3452 | Yes |
| 103 | FANCA |  |  | 199 | 1.173 | 0.3475 | Yes |
| 104 | TCEAL7 |  |  | 200 | 1.171 | 0.3503 | Yes |
| 105 | USP1 |  |  | 202 | 1.168 | 0.3529 | Yes |
| 106 | ZWINT |  |  | 204 | 1.166 | 0.3554 | Yes |
| 107 | ASPM |  |  | 207 | 1.163 | 0.3578 | Yes |
| 108 | PMP2 |  |  | 209 | 1.162 | 0.3603 | Yes |
| 109 | RBBP8 |  |  | 210 | 1.162 | 0.3631 | Yes |
| 110 | PRC1 |  |  | 211 | 1.161 | 0.3658 | Yes |
| 111 | KIF22 |  |  | 213 | 1.157 | 0.3684 | Yes |
| 112 | UBE2C |  |  | 214 | 1.154 | 0.3711 | Yes |
| 113 | PIGX |  |  | 219 | 1.150 | 0.3730 | Yes |
| 114 | CDKN2C |  |  | 223 | 1.143 | 0.3751 | Yes |
| 115 | NCAPG2 |  |  | 225 | 1.141 | 0.3776 | Yes |
| 116 | BTG3 |  |  | 226 | 1.141 | 0.3803 | Yes |
| 117 | CDC45 |  |  | 228 | 1.138 | 0.3828 | Yes |
| 118 | PPP1R14C |  |  | 229 | 1.137 | 0.3855 | Yes |
| 119 | MCUB |  |  | 230 | 1.137 | 0.3882 | Yes |
| 120 | CDK2 |  |  | 233 | 1.134 | 0.3905 | Yes |
| 121 | ASF1B |  |  | 237 | 1.130 | 0.3925 | Yes |
| 122 | MCM2 |  |  | 238 | 1.130 | 0.3952 | Yes |
| 123 | RFC3 |  |  | 239 | 1.130 | 0.3979 | Yes |
| 124 | NDC80 |  |  | 240 | 1.129 | 0.4005 | Yes |
| 125 | MCM5 |  |  | 243 | 1.127 | 0.4028 | Yes |
| 126 | SGO2 |  |  | 244 | 1.126 | 0.4055 | Yes |
| 127 | TPX2 |  |  | 248 | 1.122 | 0.4075 | Yes |
| 128 | BRIP1 |  |  | 249 | 1.121 | 0.4102 | Yes |
| 129 | SMC1A |  |  | 251 | 1.120 | 0.4126 | Yes |
| 130 | SAC3D1 |  |  | 252 | 1.118 | 0.4153 | Yes |
| 131 | TIMELESS |  |  | 259 | 1.105 | 0.4166 | Yes |
| 132 | NFATC2IP |  |  | 269 | 1.092 | 0.4174 | Yes |
| 133 | KIF14 |  |  | 272 | 1.090 | 0.4195 | Yes |
| 134 | HAUS8 |  |  | 273 | 1.089 | 0.4221 | Yes |
| 135 | BRCA2 |  |  | 274 | 1.089 | 0.4247 | Yes |
| 136 | BUB1 |  |  | 277 | 1.084 | 0.4269 | Yes |
| 137 | MCM6 |  |  | 282 | 1.080 | 0.4286 | Yes |
| 138 | CKAP2L |  |  | 285 | 1.078 | 0.4307 | Yes |
| 139 | HIRIP3 |  |  | 289 | 1.077 | 0.4327 | Yes |
| 140 | PAQR4 |  |  | 291 | 1.076 | 0.4350 | Yes |
| 141 | CDK5RAP2 |  |  | 292 | 1.076 | 0.4376 | Yes |
| 142 | PRIM1 |  |  | 293 | 1.074 | 0.4401 | Yes |
| 143 | ECT2 |  |  | 295 | 1.071 | 0.4424 | Yes |
| 144 | AURKB |  |  | 297 | 1.068 | 0.4448 | Yes |
| 145 | ARHGAP11A |  |  | 303 | 1.064 | 0.4462 | Yes |
| 146 | CCNA2 |  |  | 309 | 1.057 | 0.4477 | Yes |
| 147 | CKAP2 |  |  | 312 | 1.053 | 0.4498 | Yes |
| 148 | PSIP1 |  |  | 313 | 1.053 | 0.4523 | Yes |
| 149 | NUP210 |  |  | 316 | 1.048 | 0.4544 | Yes |
| 150 | PRR11 |  |  | 317 | 1.048 | 0.4569 | Yes |
| 151 | OXCT1 |  |  | 321 | 1.045 | 0.4587 | Yes |
| 152 | WEE1 |  |  | 322 | 1.044 | 0.4612 | Yes |
| 153 | CDC6 |  |  | 328 | 1.040 | 0.4626 | Yes |
| 154 | CDCA7 |  |  | 330 | 1.038 | 0.4649 | Yes |
| 155 | DDX11 |  |  | 331 | 1.038 | 0.4673 | Yes |
| 156 | TMEM106C |  |  | 334 | 1.034 | 0.4694 | Yes |
| 157 | RFC2 |  |  | 337 | 1.025 | 0.4714 | Yes |
| 158 | PIMREG |  |  | 339 | 1.025 | 0.4736 | Yes |
| 159 | ZGRF1 |  |  | 340 | 1.024 | 0.4760 | Yes |
| 160 | KNTC1 |  |  | 342 | 1.023 | 0.4783 | Yes |
| 161 | POLE |  |  | 344 | 1.023 | 0.4805 | Yes |
| 162 | WDR76 |  |  | 350 | 1.015 | 0.4818 | Yes |
| 163 | EXO1 |  |  | 354 | 1.010 | 0.4836 | Yes |
| 164 | CENPX |  |  | 355 | 1.010 | 0.4860 | Yes |
| 165 | RBL1 |  |  | 358 | 0.999 | 0.4880 | Yes |
| 166 | GINS4 |  |  | 359 | 0.997 | 0.4903 | Yes |
| 167 | KIF11 |  |  | 369 | 0.982 | 0.4908 | Yes |
| 168 | DNAJC9 |  |  | 373 | 0.979 | 0.4925 | Yes |
| 169 | MYBL1 |  |  | 375 | 0.977 | 0.4946 | Yes |
| 170 | DSN1 |  |  | 377 | 0.975 | 0.4967 | Yes |
| 171 | PHF19 |  |  | 378 | 0.971 | 0.4990 | Yes |
| 172 | CASP8AP2 |  |  | 379 | 0.970 | 0.5013 | Yes |
| 173 | PSMC3IP |  |  | 382 | 0.968 | 0.5032 | Yes |
| 174 | GINS1 |  |  | 385 | 0.965 | 0.5051 | Yes |
| 175 | UHRF1 |  |  | 390 | 0.958 | 0.5065 | Yes |
| 176 | ZNF367 |  |  | 394 | 0.956 | 0.5081 | Yes |
| 177 | RPA3 |  |  | 395 | 0.953 | 0.5104 | Yes |
| 178 | YEATS4 |  |  | 397 | 0.951 | 0.5125 | Yes |
| 179 | SLBP |  |  | 403 | 0.949 | 0.5137 | Yes |
| 180 | HJURP |  |  | 406 | 0.945 | 0.5155 | Yes |
| 181 | MASTL |  |  | 407 | 0.945 | 0.5177 | Yes |
| 182 | RMI1 |  |  | 411 | 0.939 | 0.5193 | Yes |
| 183 | SPC24 |  |  | 414 | 0.938 | 0.5211 | Yes |
| 184 | NUDT1 |  |  | 417 | 0.936 | 0.5229 | Yes |
| 185 | MAD2L1 |  |  | 421 | 0.932 | 0.5245 | Yes |
| 186 | KPNA2 |  |  | 435 | 0.919 | 0.5240 | Yes |
| 187 | DDIAS |  |  | 436 | 0.918 | 0.5262 | Yes |
| 188 | RACGAP1 |  |  | 443 | 0.912 | 0.5271 | Yes |
| 189 | VRK1 |  |  | 445 | 0.911 | 0.5291 | Yes |
| 190 | CENPH |  |  | 448 | 0.908 | 0.5308 | Yes |
| 191 | ORC6 |  |  | 449 | 0.908 | 0.5330 | Yes |
| 192 | STIL |  |  | 455 | 0.903 | 0.5341 | Yes |
| 193 | ZNF738 |  |  | 458 | 0.901 | 0.5358 | Yes |
| 194 | CCNE1 |  |  | 461 | 0.901 | 0.5375 | Yes |
| 195 | ECI1 |  |  | 462 | 0.901 | 0.5396 | Yes |
| 196 | POLA1 |  |  | 463 | 0.900 | 0.5418 | Yes |
| 197 | SHCBP1 |  |  | 465 | 0.900 | 0.5437 | Yes |
| 198 | POC1A |  |  | 467 | 0.896 | 0.5456 | Yes |
| 199 | MGME1 |  |  | 469 | 0.892 | 0.5475 | Yes |
| 200 | CENPJ |  |  | 479 | 0.885 | 0.5478 | Yes |
| 201 | MND1 |  |  | 480 | 0.885 | 0.5499 | Yes |
| 202 | SPAG5 |  |  | 481 | 0.884 | 0.5519 | Yes |
| 203 | BLM |  |  | 486 | 0.879 | 0.5532 | Yes |
| 204 | HUNK |  |  | 488 | 0.877 | 0.5551 | Yes |
| 205 | MPHOSPH9 |  |  | 489 | 0.877 | 0.5572 | Yes |
| 206 | ABHD3 |  |  | 494 | 0.872 | 0.5584 | Yes |
| 207 | H2AFY |  |  | 497 | 0.868 | 0.5600 | Yes |
| 208 | GMPS |  |  | 500 | 0.865 | 0.5617 | Yes |
| 209 | PAXX |  |  | 501 | 0.864 | 0.5637 | Yes |
| 210 | PKMYT1 |  |  | 506 | 0.859 | 0.5649 | Yes |
| 211 | ZNF519 |  |  | 508 | 0.857 | 0.5668 | Yes |
| 212 | BRI3BP |  |  | 510 | 0.855 | 0.5686 | Yes |
| 213 | PHIP |  |  | 513 | 0.854 | 0.5702 | Yes |
| 214 | UBE2S |  |  | 514 | 0.854 | 0.5722 | Yes |
| 215 | SAMD1 |  |  | 515 | 0.854 | 0.5742 | Yes |
| 216 | DCK |  |  | 516 | 0.854 | 0.5763 | Yes |
| 217 | TTF2 |  |  | 517 | 0.853 | 0.5783 | Yes |
| 218 | RFC5 |  |  | 527 | 0.849 | 0.5784 | Yes |
| 219 | ANKRD9 |  |  | 529 | 0.848 | 0.5802 | Yes |
| 220 | LMNB2 |  |  | 534 | 0.846 | 0.5814 | Yes |
| 221 | BUB1B |  |  | 535 | 0.846 | 0.5834 | Yes |
| 222 | BIRC5 |  |  | 541 | 0.842 | 0.5844 | Yes |
| 223 | TCF19 |  |  | 542 | 0.842 | 0.5864 | Yes |
| 224 | CEP152 |  |  | 547 | 0.834 | 0.5875 | Yes |
| 225 | SLF1 |  |  | 549 | 0.831 | 0.5893 | Yes |
| 226 | CENPQ |  |  | 557 | 0.825 | 0.5898 | Yes |
| 227 | TEDC1 |  |  | 559 | 0.825 | 0.5915 | Yes |
| 228 | WDHD1 |  |  | 562 | 0.823 | 0.5931 | Yes |
| 229 | E2F2 |  |  | 576 | 0.814 | 0.5923 | Yes |
| 230 | CMC2 |  |  | 580 | 0.812 | 0.5936 | Yes |
| 231 | DIAPH3 |  |  | 585 | 0.808 | 0.5947 | Yes |
| 232 | CENPP |  |  | 587 | 0.808 | 0.5964 | Yes |
| 233 | TK1 |  |  | 591 | 0.806 | 0.5977 | Yes |
| 234 | MSH6 |  |  | 593 | 0.804 | 0.5994 | Yes |
| 235 | RAD18 |  |  | 603 | 0.799 | 0.5994 | Yes |
| 236 | RBBP4 |  |  | 610 | 0.794 | 0.6000 | Yes |
| 237 | CDCA4 |  |  | 615 | 0.790 | 0.6011 | Yes |
| 238 | RFWD3 |  |  | 616 | 0.790 | 0.6029 | Yes |
| 239 | E2F7 |  |  | 618 | 0.789 | 0.6046 | Yes |
| 240 | GEN1 |  |  | 619 | 0.789 | 0.6065 | Yes |
| 241 | HMMR |  |  | 622 | 0.788 | 0.6079 | Yes |
| 242 | CDC25A |  |  | 631 | 0.782 | 0.6081 | Yes |
| 243 | IPO9 |  |  | 639 | 0.779 | 0.6085 | Yes |
| 244 | CENPL |  |  | 640 | 0.778 | 0.6104 | Yes |
| 245 | NUP62 |  |  | 664 | 0.767 | 0.6074 | Yes |
| 246 | SKA3 |  |  | 668 | 0.765 | 0.6086 | Yes |
| 247 | WDR34 |  |  | 671 | 0.762 | 0.6100 | Yes |
| 248 | FANCB |  |  | 681 | 0.759 | 0.6099 | Yes |
| 249 | CENPO |  |  | 686 | 0.755 | 0.6109 | Yes |
| 250 | ZNF726 |  |  | 694 | 0.748 | 0.6112 | Yes |
| 251 | KIF18B |  |  | 698 | 0.747 | 0.6123 | Yes |
| 252 | ARL6IP6 |  |  | 702 | 0.745 | 0.6135 | Yes |
| 253 | UNG |  |  | 703 | 0.745 | 0.6152 | Yes |
| 254 | SMC3 |  |  | 708 | 0.743 | 0.6162 | Yes |
| 255 | TFDP1 |  |  | 711 | 0.742 | 0.6175 | Yes |
| 256 | FIGNL1 |  |  | 714 | 0.741 | 0.6189 | Yes |
| 257 | NETO2 |  |  | 717 | 0.740 | 0.6202 | Yes |
| 258 | PLK4 |  |  | 720 | 0.739 | 0.6215 | Yes |
| 259 | ATAD5 |  |  | 721 | 0.739 | 0.6233 | Yes |
| 260 | KDELC2 |  |  | 723 | 0.738 | 0.6248 | Yes |
| 261 | MAD2L2 |  |  | 724 | 0.736 | 0.6266 | Yes |
| 262 | PGP |  |  | 727 | 0.732 | 0.6279 | Yes |
| 263 | HAT1 |  |  | 741 | 0.727 | 0.6269 | Yes |
| 264 | USP37 |  |  | 751 | 0.723 | 0.6268 | Yes |
| 265 | NABP2 |  |  | 769 | 0.714 | 0.6249 | Yes |
| 266 | TUBG1 |  |  | 776 | 0.712 | 0.6254 | Yes |
| 267 | CHEK1 |  |  | 779 | 0.709 | 0.6266 | Yes |
| 268 | ACTL6A |  |  | 780 | 0.709 | 0.6283 | Yes |
| 269 | CCDC15 |  |  | 782 | 0.709 | 0.6298 | Yes |
| 270 | CBX5 |  |  | 783 | 0.708 | 0.6315 | Yes |
| 271 | RAD54L |  |  | 790 | 0.704 | 0.6319 | Yes |
| 272 | CEP78 |  |  | 791 | 0.704 | 0.6336 | Yes |
| 273 | HPF1 |  |  | 796 | 0.702 | 0.6344 | Yes |
| 274 | CD320 |  |  | 799 | 0.701 | 0.6356 | Yes |
| 275 | DTYMK |  |  | 800 | 0.700 | 0.6373 | Yes |
| 276 | TRIP13 |  |  | 804 | 0.698 | 0.6383 | Yes |
| 277 | RSRC1 |  |  | 818 | 0.693 | 0.6373 | Yes |
| 278 | RBBP7 |  |  | 829 | 0.687 | 0.6368 | Yes |
| 279 | LRRC45 |  |  | 831 | 0.687 | 0.6383 | Yes |
| 280 | ALYREF |  |  | 847 | 0.680 | 0.6367 | Yes |
| 281 | PCNT |  |  | 857 | 0.675 | 0.6365 | Yes |
| 282 | RNASEH2A |  |  | 859 | 0.674 | 0.6379 | Yes |
| 283 | PARP2 |  |  | 864 | 0.673 | 0.6386 | Yes |
| 284 | DDB2 |  |  | 872 | 0.669 | 0.6388 | Yes |
| 285 | NUP50 |  |  | 875 | 0.668 | 0.6399 | Yes |
| 286 | CHEK2 |  |  | 882 | 0.667 | 0.6403 | Yes |
| 287 | C19orf48 |  |  | 893 | 0.661 | 0.6398 | Yes |
| 288 | IMPA2 |  |  | 903 | 0.659 | 0.6394 | Yes |
| 289 | PPP2R3B |  |  | 907 | 0.658 | 0.6404 | Yes |
| 290 | MELK |  |  | 915 | 0.653 | 0.6405 | Yes |
| 291 | MZT1 |  |  | 917 | 0.652 | 0.6418 | Yes |
| 292 | KIF20B |  |  | 934 | 0.647 | 0.6400 | Yes |
| 293 | PTTG1 |  |  | 936 | 0.647 | 0.6413 | Yes |
| 294 | HMGXB4 |  |  | 943 | 0.645 | 0.6416 | Yes |
| 295 | TMEM237 |  |  | 952 | 0.639 | 0.6415 | Yes |
| 296 | CENPN |  |  | 953 | 0.638 | 0.6430 | Yes |
| 297 | TMEM97 |  |  | 963 | 0.636 | 0.6426 | Yes |
| 298 | PEG10 |  |  | 964 | 0.636 | 0.6441 | Yes |
| 299 | CKS2 |  |  | 966 | 0.634 | 0.6454 | Yes |
| 300 | PRIM2 |  |  | 968 | 0.633 | 0.6467 | Yes |
| 301 | CLN6 |  |  | 975 | 0.630 | 0.6470 | Yes |
| 302 | C1orf21 |  |  | 980 | 0.627 | 0.6476 | Yes |
| 303 | HAUS1 |  |  | 987 | 0.624 | 0.6479 | Yes |
| 304 | ZNF93 |  |  | 988 | 0.624 | 0.6493 | Yes |
| 305 | LRR1 |  |  | 991 | 0.623 | 0.6504 | Yes |
| 306 | POLD1 |  |  | 998 | 0.620 | 0.6506 | Yes |
| 307 | CASP2 |  |  | 1001 | 0.620 | 0.6517 | Yes |
| 308 | LSM4 |  |  | 1003 | 0.619 | 0.6529 | Yes |
| 309 | RABL6 |  |  | 1022 | 0.615 | 0.6507 | Yes |
| 310 | MIS18A |  |  | 1023 | 0.614 | 0.6521 | Yes |
| 311 | NRM |  |  | 1031 | 0.613 | 0.6521 | Yes |
| 312 | MNS1 |  |  | 1034 | 0.613 | 0.6531 | Yes |
| 313 | PAFAH1B3 |  |  | 1039 | 0.612 | 0.6538 | Yes |
| 314 | CDCA8 |  |  | 1043 | 0.610 | 0.6546 | Yes |
| 315 | ZWILCH |  |  | 1048 | 0.609 | 0.6552 | Yes |
| 316 | CEP57 |  |  | 1053 | 0.607 | 0.6558 | Yes |
| 317 | CDC7 |  |  | 1060 | 0.604 | 0.6560 | Yes |
| 318 | TTF1 |  |  | 1080 | 0.597 | 0.6535 | Yes |
| 319 | DONSON |  |  | 1082 | 0.597 | 0.6547 | Yes |
| 320 | SRSF2 |  |  | 1084 | 0.597 | 0.6559 | Yes |
| 321 | PHTF2 |  |  | 1094 | 0.594 | 0.6554 | Yes |
| 322 | NAP1L4 |  |  | 1106 | 0.590 | 0.6545 | Yes |
| 323 | MSH2 |  |  | 1107 | 0.590 | 0.6559 | Yes |
| 324 | VMA21 |  |  | 1112 | 0.589 | 0.6565 | Yes |
| 325 | LHX2 |  |  | 1121 | 0.587 | 0.6562 | Yes |
| 326 | SMCHD1 |  |  | 1123 | 0.587 | 0.6574 | Yes |
| 327 | TEDC2 |  |  | 1142 | 0.579 | 0.6550 | Yes |
| 328 | HMGB3 |  |  | 1154 | 0.577 | 0.6541 | Yes |
| 329 | POLA2 |  |  | 1161 | 0.574 | 0.6542 | Yes |
| 330 | EME1 |  |  | 1165 | 0.573 | 0.6550 | Yes |
| 331 | DEPDC1 |  |  | 1179 | 0.569 | 0.6536 | Yes |
| 332 | ITGB3BP |  |  | 1180 | 0.568 | 0.6550 | Yes |
| 333 | SPIN4 |  |  | 1190 | 0.565 | 0.6544 | Yes |
| 334 | MEA1 |  |  | 1197 | 0.562 | 0.6545 | Yes |
| 335 | POLE2 |  |  | 1199 | 0.561 | 0.6556 | Yes |
| 336 | PARPBP |  |  | 1210 | 0.560 | 0.6549 | Yes |
| 337 | TMEM54 |  |  | 1215 | 0.559 | 0.6554 | Yes |
| 338 | POLR2D |  |  | 1220 | 0.556 | 0.6559 | Yes |
| 339 | CBR3 |  |  | 1221 | 0.556 | 0.6572 | Yes |
| 340 | EXOSC8 |  |  | 1230 | 0.553 | 0.6568 | Yes |
| 341 | RAD1 |  |  | 1234 | 0.552 | 0.6575 | Yes |
| 342 | HADH |  |  | 1242 | 0.548 | 0.6574 | Yes |
| 343 | SPATA33 |  |  | 1251 | 0.546 | 0.6570 | Yes |
| 344 | CTDSPL2 |  |  | 1256 | 0.544 | 0.6575 | Yes |
| 345 | CSE1L |  |  | 1259 | 0.543 | 0.6583 | Yes |
| 346 | RMI2 |  |  | 1261 | 0.542 | 0.6594 | Yes |
| 347 | SRSF10 |  |  | 1289 | 0.535 | 0.6551 | No |
| 348 | ICMT |  |  | 1298 | 0.532 | 0.6547 | No |
| 349 | CCDC167 |  |  | 1303 | 0.531 | 0.6551 | No |
| 350 | RPA1 |  |  | 1307 | 0.530 | 0.6557 | No |
| 351 | FOXRED2 |  |  | 1308 | 0.530 | 0.6570 | No |
| 352 | KIF2C |  |  | 1309 | 0.530 | 0.6582 | No |
| 353 | MRE11 |  |  | 1335 | 0.523 | 0.6543 | No |
| 354 | RAC3 |  |  | 1339 | 0.523 | 0.6549 | No |
| 355 | DIAPH1 |  |  | 1344 | 0.522 | 0.6553 | No |
| 356 | CMSS1 |  |  | 1350 | 0.520 | 0.6555 | No |
| 357 | MORC4 |  |  | 1352 | 0.519 | 0.6565 | No |
| 358 | FANCL |  |  | 1353 | 0.519 | 0.6577 | No |
| 359 | CCNB2 |  |  | 1373 | 0.513 | 0.6550 | No |
| 360 | PDLIM4 |  |  | 1382 | 0.511 | 0.6546 | No |
| 361 | ERCC6L |  |  | 1383 | 0.511 | 0.6558 | No |
| 362 | NUP107 |  |  | 1388 | 0.509 | 0.6561 | No |
| 363 | SPDL1 |  |  | 1397 | 0.507 | 0.6557 | No |
| 364 | YWHAH |  |  | 1407 | 0.502 | 0.6550 | No |
| 365 | SLC25A40 |  |  | 1408 | 0.502 | 0.6562 | No |
| 366 | GGCT |  |  | 1414 | 0.501 | 0.6563 | No |
| 367 | SHMT1 |  |  | 1417 | 0.499 | 0.6571 | No |
| 368 | NUDT8 |  |  | 1424 | 0.498 | 0.6570 | No |
| 369 | ORC1 |  |  | 1447 | 0.493 | 0.6536 | No |
| 370 | CNPY3 |  |  | 1453 | 0.492 | 0.6538 | No |
| 371 | TAF15 |  |  | 1463 | 0.490 | 0.6531 | No |
| 372 | ZNF730 |  |  | 1467 | 0.488 | 0.6536 | No |
| 373 | EXOSC9 |  |  | 1472 | 0.487 | 0.6539 | No |
| 374 | FN3KRP |  |  | 1474 | 0.487 | 0.6549 | No |
| 375 | POLR3K |  |  | 1476 | 0.487 | 0.6558 | No |
| 376 | CKLF |  |  | 1480 | 0.486 | 0.6563 | No |
| 377 | ALDH7A1 |  |  | 1496 | 0.481 | 0.6544 | No |
| 378 | RFC1 |  |  | 1499 | 0.480 | 0.6551 | No |
| 379 | SUPT16H |  |  | 1500 | 0.480 | 0.6562 | No |
| 380 | CKS1B |  |  | 1502 | 0.479 | 0.6571 | No |
| 381 | CHTF18 |  |  | 1507 | 0.479 | 0.6575 | No |
| 382 | CEP55 |  |  | 1508 | 0.479 | 0.6586 | No |
| 383 | C5orf34 |  |  | 1514 | 0.478 | 0.6587 | No |
| 384 | NUP85 |  |  | 1529 | 0.475 | 0.6569 | No |
| 385 | ABHD11 |  |  | 1534 | 0.473 | 0.6572 | No |
| 386 | KHK |  |  | 1544 | 0.471 | 0.6564 | No |
| 387 | SFI1 |  |  | 1553 | 0.470 | 0.6559 | No |
| 388 | SRRT |  |  | 1563 | 0.467 | 0.6551 | No |
| 389 | POP7 |  |  | 1591 | 0.461 | 0.6506 | No |
| 390 | UBA2 |  |  | 1612 | 0.457 | 0.6475 | No |
| 391 | SASS6 |  |  | 1618 | 0.457 | 0.6476 | No |
| 392 | SSRP1 |  |  | 1634 | 0.451 | 0.6455 | No |
| 393 | HDGFL3 |  |  | 1654 | 0.445 | 0.6426 | No |
| 394 | TPGS2 |  |  | 1663 | 0.444 | 0.6420 | No |
| 395 | SLC2A4RG |  |  | 1683 | 0.440 | 0.6391 | No |
| 396 | NR2C2AP |  |  | 1690 | 0.438 | 0.6389 | No |
| 397 | PRPS2 |  |  | 1702 | 0.435 | 0.6376 | No |
| 398 | THOP1 |  |  | 1722 | 0.433 | 0.6347 | No |
| 399 | DERA |  |  | 1749 | 0.428 | 0.6303 | No |
| 400 | SLC25A10 |  |  | 1783 | 0.422 | 0.6245 | No |
| 401 | DCTPP1 |  |  | 1796 | 0.418 | 0.6229 | No |
| 402 | SNX5 |  |  | 1849 | 0.410 | 0.6131 | No |
| 403 | FKBP5 |  |  | 1893 | 0.405 | 0.6051 | No |
| 404 | NCAPH2 |  |  | 1915 | 0.401 | 0.6017 | No |
| 405 | CENPW |  |  | 1959 | 0.395 | 0.5937 | No |
| 406 | ACOT7 |  |  | 1967 | 0.394 | 0.5932 | No |
| 407 | CCND3 |  |  | 1991 | 0.390 | 0.5893 | No |
| 408 | CNTLN |  |  | 2004 | 0.387 | 0.5877 | No |
| 409 | TMX1 |  |  | 2012 | 0.386 | 0.5872 | No |
| 410 | PPM1G |  |  | 2017 | 0.385 | 0.5873 | No |
| 411 | MANF |  |  | 2023 | 0.384 | 0.5872 | No |
| 412 | CBFB |  |  | 2035 | 0.382 | 0.5858 | No |
| 413 | XPO1 |  |  | 2091 | 0.374 | 0.5752 | No |
| 414 | CDC5L |  |  | 2115 | 0.372 | 0.5713 | No |
| 415 | ANLN |  |  | 2125 | 0.371 | 0.5703 | No |
| 416 | CFAP20 |  |  | 2134 | 0.370 | 0.5695 | No |
| 417 | NUP205 |  |  | 2169 | 0.366 | 0.5633 | No |
| 418 | GPD2 |  |  | 2222 | 0.359 | 0.5534 | No |
| 419 | RAD51D |  |  | 2266 | 0.355 | 0.5453 | No |
| 420 | NUP155 |  |  | 2313 | 0.347 | 0.5365 | No |
| 421 | AEN |  |  | 2315 | 0.347 | 0.5372 | No |
| 422 | SKP2 |  |  | 2391 | 0.336 | 0.5223 | No |
| 423 | PAICS |  |  | 2403 | 0.334 | 0.5209 | No |
| 424 | HPRT1 |  |  | 2404 | 0.334 | 0.5216 | No |
| 425 | COA1 |  |  | 2406 | 0.334 | 0.5222 | No |
| 426 | CHCHD3 |  |  | 2434 | 0.330 | 0.5174 | No |
| 427 | SKA2 |  |  | 2445 | 0.329 | 0.5161 | No |
| 428 | LKAAEAR1 |  |  | 2460 | 0.327 | 0.5140 | No |
| 429 | PPIF |  |  | 2478 | 0.325 | 0.5112 | No |
| 430 | SDC1 |  |  | 2491 | 0.323 | 0.5095 | No |
| 431 | BID |  |  | 2534 | 0.319 | 0.5015 | No |
| 432 | RPA2 |  |  | 2570 | 0.314 | 0.4950 | No |
| 433 | AHCY |  |  | 2598 | 0.311 | 0.4901 | No |
| 434 | MRM2 |  |  | 2622 | 0.307 | 0.4860 | No |
| 435 | COLGALT1 |  |  | 2623 | 0.307 | 0.4868 | No |
| 436 | CCHCR1 |  |  | 2644 | 0.305 | 0.4833 | No |
| 437 | DCXR |  |  | 2647 | 0.305 | 0.4836 | No |
| 438 | C1orf35 |  |  | 2665 | 0.303 | 0.4808 | No |
| 439 | RTN3 |  |  | 2671 | 0.302 | 0.4805 | No |
| 440 | JPT1 |  |  | 2712 | 0.298 | 0.4729 | No |
| 441 | SNRPA |  |  | 2738 | 0.296 | 0.4684 | No |
| 442 | MESP1 |  |  | 2754 | 0.294 | 0.4660 | No |
| 443 | BUB3 |  |  | 2806 | 0.288 | 0.4560 | No |
| 444 | PRADC1 |  |  | 2845 | 0.285 | 0.4488 | No |
| 445 | GINS3 |  |  | 2847 | 0.284 | 0.4493 | No |
| 446 | SAE1 |  |  | 2872 | 0.283 | 0.4449 | No |
| 447 | PMF1 |  |  | 2895 | 0.279 | 0.4410 | No |
| 448 | MTHFD1 |  |  | 2917 | 0.277 | 0.4373 | No |
| 449 | CNOT9 |  |  | 2951 | 0.274 | 0.4311 | No |
| 450 | APOO |  |  | 3012 | 0.268 | 0.4193 | No |
| 451 | CHAF1B |  |  | 3047 | 0.264 | 0.4128 | No |
| 452 | DPM2 |  |  | 3082 | 0.261 | 0.4064 | No |
| 453 | RCCD1 |  |  | 3103 | 0.260 | 0.4028 | No |
| 454 | VOPP1 |  |  | 3125 | 0.258 | 0.3991 | No |
| 455 | ACD |  |  | 3131 | 0.257 | 0.3986 | No |
| 456 | ATP1B3 |  |  | 3158 | 0.254 | 0.3938 | No |
| 457 | HSPB11 |  |  | 3163 | 0.254 | 0.3936 | No |
| 458 | HAUS2 |  |  | 3186 | 0.251 | 0.3896 | No |
| 459 | MRPL22 |  |  | 3353 | -0.275 | 0.3558 | No |
| 460 | JPT2 |  |  | 3459 | -0.292 | 0.3346 | No |
| 461 | MAP3K20 |  |  | 3668 | -0.326 | 0.2921 | No |
| 462 | SOD3 |  |  | 3708 | -0.332 | 0.2848 | No |
| 463 | CAD |  |  | 3792 | -0.348 | 0.2684 | No |
| 464 | ATAD3A |  |  | 3894 | -0.366 | 0.2482 | No |
| 465 | TUBB6 |  |  | 3904 | -0.368 | 0.2472 | No |
| 466 | TUBA1C |  |  | 3957 | -0.379 | 0.2373 | No |
| 467 | SNRPF |  |  | 4037 | -0.397 | 0.2218 | No |
| 468 | ATIC |  |  | 4088 | -0.407 | 0.2124 | No |
| 469 | SHMT2 |  |  | 4131 | -0.417 | 0.2046 | No |
| 470 | NT5C3A |  |  | 4190 | -0.435 | 0.1936 | No |
| 471 | NPW |  |  | 4199 | -0.439 | 0.1930 | No |
| 472 | MTHFD2 |  |  | 4212 | -0.444 | 0.1915 | No |
| 473 | PLP2 |  |  | 4366 | -0.490 | 0.1609 | No |
| 474 | EBP |  |  | 4441 | -0.517 | 0.1467 | No |
| 475 | PSMB9 |  |  | 4503 | -0.543 | 0.1353 | No |
| 476 | PSMG1 |  |  | 4573 | -0.574 | 0.1223 | No |
| 477 | NTM |  |  | 4695 | -0.643 | 0.0987 | No |
| 478 | LRRC59 |  |  | 4749 | -0.677 | 0.0893 | No |
| 479 | MT2A |  |  | 4799 | -0.714 | 0.0808 | No |
| 480 | SMS |  |  | 4842 | -0.750 | 0.0738 | No |
| 481 | NDUFAF2 |  |  | 4897 | -0.808 | 0.0645 | No |
| 482 | MIF |  |  | 4900 | -0.811 | 0.0660 | No |
| 483 | C12orf75 |  |  | 4940 | -0.858 | 0.0599 | No |
| 484 | HMGA1 |  |  | 4962 | -0.878 | 0.0576 | No |
| 485 | SPHK1 |  |  | 4997 | -0.928 | 0.0528 | No |
| 486 | DUSP23 |  |  | 5003 | -0.941 | 0.0540 | No |
| 487 | SLC1A5 |  |  | 5145 | -1.294 | 0.0277 | No |
| 488 | PHLDA2 |  |  | 5163 | -1.384 | 0.0275 | No |
Table: GSEA details [plain text format]

  

Fig 2: DOROSZKO\_U3054MG\_CLUSTER\_4: Random ES distribution      
 Gene set null distribution of ES for **DOROSZKO\_U3054MG\_CLUSTER\_4**
